# Continuing Professional Development – Medical Imaging

**DOI:** 10.1002/jmrs.645

**Published:** 2023-01-18

Maximise your CPD by reading the following selected article and answer the five questions. Please remember to self‐claim your CPD and retain your supporting evidence. Answers will be available via the QR code and online at www.asmirt.org/news-and-publications/jmrs, as well as published in JMRS – Volume 70, Issue 4, December 2023.

## Medical Imaging – Original Article

### Determination of hepatic extraction fraction with gadoxetate low‐temporal resolution DCE‐MRI‐based deconvolution analysis: validation with ALBI score and Child‐Pugh class

Phonlakrai M, Ramadan S, Simpson J, Gholizadeh N, Arm J, Skehan K, Goodwin J, Trada Y, Martin J, Sridharan S, Lamichhane B, Bollipo S, Greer P. (2023) *J Med Radiat Sci*. 10.1002/jmrs.617

1. What do the authors of this study suggest gadoxetate MRI‐based liver function mapping could be used for in future investigations?
  - Predicting tumour response following liver radiation therapy
  - Replacing CT simulation
  - Allow visualisation for highly accurate gross tumour volume contouring
  - Functional avoidance in radiation therapy
2. Which of the following is *most* correct?
  - Hepatic extraction fraction represents hepatocyte function efficacy
  - Extracellular contrast agent is required for assessing hepatic extraction fraction
  - Pharmacokinetic tissue‐compartment modelling does not require variable optimisation
  - Deconvolution analysis method also requires the definition of tissue compartments
3. Which of the following was the limitation of clinical scoring systems in measuring liver function compared with image‐based liver function?
  - Provides only global liver function
  - Provides only regional liver function
  - Not a reliable measure in predicting mortality
  - Not widely used in routine clinical practice
4. Which of the following is *most* correct about gadoxetate liver MRI scan in routine clinical practice?
  - The patient can breathe freely during the scan
  - Low‐temporal resolution DCE‐MRI
  - High‐temporal resolution DCE‐MRI
  - The image acquisition time normally takes at least 90 min following gadoxetate administration
5. Which of the following is correct regarding hepatic extraction fraction (HEF)?
  - Image‐based HEF correlated with the clinical scoring system (ALBI score)
  - HEF had a high variability and was unacceptable
  - HEF could distinguish between non‐cirrhosis and cirrhosis patients
  - Both a. and c. are correct

### Recommended further reading:

1. Wan S‐Z, Nie Y, Zhang Y, Liu C, Zhu X. Assessing the prognostic performance of the child‐pugh, model for end‐stage liver disease, and albumin‐bilirubin scores in patients with decompensated cirrhosis: a large asian cohort from gastroenterology department. *Dis Markers* 2020; 2020: 5193028.
2. Nilsson H, Nordell A, Vargas R, Douglas L, Jonas E, Blomqvist L. Assessment of hepatic extraction fraction and input relative blood flow using dynamic hepatocyte‐specific contrast‐enhanced MRI. *J Magn Reson Imaging* 2009; 29: 1323–31.
3. Wang H, Cao Y. Spatially resolved assessment of hepatic function using 99mTc‐IDA SPECT. *Med Phys* 2013; 40: 92501.

## Answers


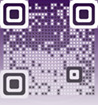


Scan this QR code to find the answers, or visit www.asmirt.org/news-and-publications/jmrs

